# Recent Biomedical Applications of Coupling Nanocomposite Polymeric Materials Reinforced with Variable Carbon Nanofillers

**DOI:** 10.3390/biomedicines11030967

**Published:** 2023-03-21

**Authors:** Abeer M. Alosaimi, Randa O. Alorabi, Dina F. Katowah, Zahrah T. Al-Thagafi, Eman S. Alsolami, Mahmoud A. Hussein, Mohammad Qutob, Mohd Rafatullah

**Affiliations:** 1 Department of Chemistry, Faculty of Science, Taif University, P.O. Box 11099, Taif 21944, Saudi Arabia; 2 Chemistry Department, Faculty of Science, Ibb University, Ibb 70270, Yemen; 3 Department of Chemistry, Faculty of Applied Science, Umm Al-Qura University, P.O. Box 16722, Makkah 21955, Saudi Arabia; 4 Chemistry Department, Faculty of Science, King Abdulaziz University, P.O. Box 80203, Jeddah 21589, Saudi Arabia; 5 Chemistry Department, Faculty of Science, Assiut University, Assiut 71516, Egypt; 6 Environmental Technology Division, School of Industrial Technology, Universiti Sains Malaysia, Penang 11800, Malaysia; 7 Green Biopolymer, Coatings & Packaging Cluster, School of Industrial Technology, Universiti Sains Malaysia, Penang 11800, Malaysia

**Keywords:** hybrid materials, biomaterials, biomedical applications, carbon-based materials, bio-nanocomposites

## Abstract

The hybridization between polymers and carbon materials is one of the most recent and crucial study areas which abstracted more concern from scientists in the past few years. Polymers could be classified into two classes according to the source materials synthetic and natural. Synthetic polymeric materials have been applied over a floppy zone of industrial fields including the field of biomedicine. Carbon nanomaterials including (fullerene, carbon nanotubes, and graphene) classified as one of the most significant sources of hybrid materials. Nanocarbons are improving significantly mechanical properties of polymers in nanocomposites in addition to physical and chemical properties of the new materials. In all varieties of proposed bio-nanocomposites, a considerable improvement in the microbiological performance of the materials has been explored. Various polymeric materials and carbon-course nanofillers were present, along with antibacterial, antifungal, and anticancer products. This review spots the light on the types of synthetic polymers-based carbon materials and presented state-of-art examples on their application in the area of biomedicine.

## 1. Introduction

Polymers are an affordable material, easy to perform, and a crucial set of materials for today’s environment. The polymers are contained from a series of single units called monomers, which total indicates the level of polymerization. Polymers could be classified based on parent materials (natural or synthetic), chemical makeup (organic or inorganic), type of monomer unit (homopolymers or copolymers), signs of degradation (chemical, biological, etc.), stability (e.g., thermal, mechanical, etc.), and applications of polymers are all used to categorize them [1]. Due to their unique characteristics of polymers, researchers from various fields of science are drawn to apply in their areas. Since roughly 50 years ago, polymeric biomaterials have been a multidisciplinary and interdisciplinary field, and their development has been influenced by developments in chemistry, biology, medicine, physics, and materials [2]. The generation of biomimetic polymer hybrid materials with hierarchical structures at all scales, and the approximate of biological analogues in terms of dynamic and adjustable features is the main goal of researchers working in this field. The interactivity is crucial for maximizing production, enhancing performance, and achieving the necessary attributes for each application’s specific aims [3]. A vast range of biomedical polymers have been applied in a different biomedical field including surgical sutures and implants. This widespread use of Biomedical polymers due not only to their variety, adaptability, and affordable price, but also to the chemical compositions and flexibility, which gives them different chemical, biological, and physical behavior and improves their processability and manufacturing potential. Despite the numerous improvements in polymer science, there is no single polymer can accomplish everything. Particularly in biological applications, their low mechanical strength is a problem. This is what drives the development of innovative, polymer-based biomaterials with a range of functional groups. Natural polymers with excellent structural characteristics are used to create synthetic biomaterials. The biopolymer gives its distinctive characteristics or intense variable interactions, and the preparative ingredient offers responsiveness, structural stability, and low cost. Self-assembly of synthetic building blocks, biomacromolecules, and in particular polymers with supramolecular and dynamic properties has all produced the distinctive bioinspired or biomimetic components [4] creating polymers with various structural configurations, such as composites, networks, mixes, and copolymers. Access to a wide range of biomaterials with desirable developments is made possible by all of these forms.

## 2. Synthetic Polymers

It has been appreciated by huge number of researches in biomedicine because its unbelievable customizable characters which include: porosity, degradation time, and mechanical performances. They tend to be less expensive compared to natural polymers; might orderly prepared in bigger batches; and have a longer shelf-life. However, one major limitation of synthetic polymers is their inability to generate biological signals to initiate cell adhesion, proliferation, and tissue recovery. Enhancing the biologically useful properties of these polymers and to promote their interconnections with cells, hybrid materials in the form of nanocomposites composed of both types of polymers totally synthetic or modified natural additionally to the biodegradable polymers have been determined in details [5]. Under carefully controlled conditions, the mechanical and physical characters of polymers, including the tensile strength, elastic modulus, and the rate of decomposition, can be tailored. Typically, polymers are polydisperse and are produced with controllable limits over their architecture and functional groups. For biomedical applications, chosen of the material is based on the degree of inertness and how well it can mimic the physical characters of the harmed tissue.

**Figure 1:**
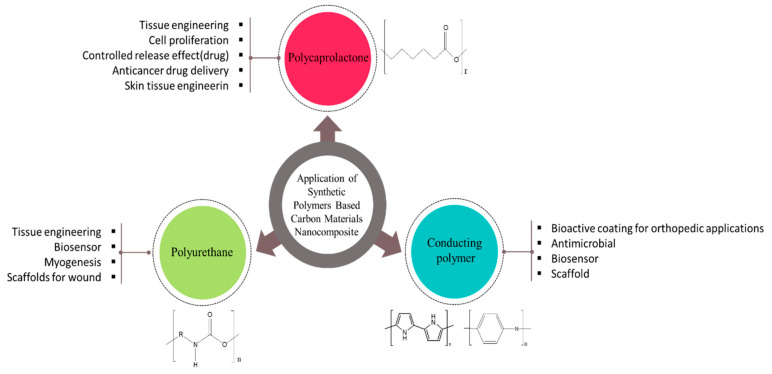
Schematic illustration for the most common synthetic polymers for biomedical applications.

A state-of-art techniques and strategies have been used for the combination between such as reversible addition fragmentation chain transfer polymerization, ring-opening-mediated radical polymerization, and atom-transfer radical polymerization. These strategies have been successful for combining synthetic and natural polymers to obtain unique multifunctional and/or nanostructured materials [5]. Nowadays, hybrid compounds continuing such a smart, stimuli-responsive polymeric materials have become popular as results of their capability to react with tiny changes in temperature, pH, light, and electric or magnetic fields, allowing for some important applications specially in tissue engineering, drug delivery, bio-separation, and biosensor designing. Supramolecular polymeric materials known as dynamers, dynamic polymers, or adaptamers, possess noncovalent interactions and or dynamic, reversible covalent bonds between complexes, allowing them to change their structures in response to various physico-chemical stimuli. Dynamats also known as “dynamers” are using for susceptible, self-healing, and adaptive biomaterials synthesized and formed via spontaneous process self-organization as the formation of reversible Diels-Alder reactions and reversible covalent bonds. Figure 1 is a simple schematic representation of the most important types of synthetic polymers for biomedical applications.

### 2.1. Synthetic Biostable Polymers

In the field of medicine, synthetic polymers based on biostable materials have been utilized for a higher range of time and introduced in medical industries to utilize in a different field of medicine such as infusion pumps, intra-aortic balloons, vascular prostheses, endotracheal tubes, catheters, and cannulas, foams, films, tissue-engineering scaffolds, and nonabsorbable surgical sutures. The majority of synthetic polymers are stable and nonreactive under regular physiological settings. This is because the internal chemical linkages are required to be of sufficient strength to maintain the compound’s integrity. The bio-decomposition pathway of synthetic polymers are deeply dependent on parent materials and chemical structure, which participated to synthesized the polymers with partially biodegradable materials [5]. This is because their biodegradation pathways are started referring to as their chemical structure and constituents. Some examples of synthetic, biostable polymers are as follows: polyethylenes, polypropylenes, polytetrafluoroethylene, poly(meth)acrylates, polyacrylamides, poly(*N*-isopropylacrylamide), polyethylene glycol, poly(ethylene oxide), polyethylene terephthalate, polyamides, polyether ether ketone, and polyurethanes.

### 2.2. Synthetic Biodegradable Polymers

The hydrolyzable bonds found in synthetic biodegradable polymers come in the form of esters, anhydrides, carbonates, and amides. These hydrolyzable bonds disintegrate within the body as a consequence of chemical interactions as well as physical and biological processes. Surface or bulk biodegradation might take place throughout the process of biodegradation. Degradation of the hydrophobic polymer surface may take place while the inside structure is maintained; this gives hydrophobic polymers better control over the pace of surface degradation. Because the process of degradation takes place over the whole of the material’s volume, the biomaterial will disintegrate as a result of the fast absorption of water by hydrophilic polymers during bulk degradation. This will lead the biomaterial to fail. When polymer biomaterials come in contact with bodily fluids, in the first step, water-labile link created when biomaterial absorbs water and expands to touch with the polymeric substances. Adjusting the proportion of hydrophobic monomers to hydrophilic monomers in a copolymer enables one to shape the interactions that take place between polymers and water. Controlling the intensity to which crystallization occurs is another tactic that may be used. Crystalline areas often resist the entrance of water molecules, which results in a less level of swelling. When it comes to polymer hydrogels, increasing the density of the cross-linking network is an efficient method for reducing swelling. The hydrolytic degradation of polymers could be accomplished using a variety of compounds such as enzymes, salts, acids, and bases. This type of decomposition entailed the arbitrary cleavage of linker bounds in the polymer chain by assaulting water molecules, which results in a decrease in the molecular weight. Due to the equilibrium between the kinetics of depolymerization and water uptake acting to determine the decomposition pathway ether from the surface or throughout the polymer’s volume. Both of these mechanisms are examples of how a polymer can degrade (bulk degradation mechanism), comparatively instances of synthetic biodegradable polymers for example: aliphatic polyesters, poly(glycolic acid), poly(lactic) acid, poly(lactide-*co*-glycolide), poly(ε-caprolactone), poly(propylene fumarate), polyanhydrides, and polycarbonates [6].

## 3. An Illustration of Common Synthetic Polymers

### 3.1. Poly (caprolactone)

Poly (caprolactone) (PCL) is a polyester from the aliphatic type that is biodegradable and has achieved wide use as a biomaterial in prosthetics, sutures, and drug delivery systems. According to the many advantages of PCL like the simple extrusion processing, the easy manufacturing in a different forms and structures, mild undesirable host reactions, relatively low cost, in addition the FDA permission for human utility, thus leading to increase in their demand [7,8]. PCL’s three-dimensional (3D) and directional porous structure can also be transformed into filaments for subsequent textile fabrication. PCL and its copolymers have been studied in fiber form (fiber diameters ranging from nanometers to millimeters) for use in drug delivery systems [9], “long-lasting” absorbable sutures [10,11,12,13], and 3D scaffolds for tissue engineering [14]. Caprolactone has been copolymerized with DL-lactide and trimethylene carbonate for use in absorbable nerve guides [15,16].

### 3.2. Conducting Polymers

Conductor materials specially conducting polymers have a good conductivity to weight ratio, which allows for great participant of the electrical stimulus, has good electrical and optical characteristics, and has good electrical characters. In addition, they may be made to be biocompatible, biodegradable, and permeable via the construction process. By adding antibodies, enzymes, and many other biological moieties, their chemical, electrical, and physical characteristics may also be tuned to their particular use [17,18,19]. This is accomplished by applying external stimuli such as electricity, light, or pH changes (even after synthesis) [20,21,22]. In this review, we present up-to-date information available for the most commonly used conductive polymers in medical applications.

### 3.3. Polypyrrole

Polypyrrole (PPy) has a number of advantageous properties, including stimuli-responsive properties, making it a forthcoming developed biomaterial [21,23]. Most notably, it possesses good biocompatibility both in vitro and in vivo [18,24,25]; strong chemical permanence in air and water [26,27]; and a conductivity that is tolerably high under physiological circumstances [18,24,28,29]. PPy could be smoothly prepared in large quantities in a variety of solvents, including water, at room temperature [24,25,26,27,30,31,32]. It can also have diverse characteristics likre a huge surface area, and it can be altered by adding bioactive compounds for biological uses. [18,19,20,21,22,23,24,28,29,30,31,33,34]. PPy is additionally electrically responsive, allowing for control of its properties [23,35]. Unfortunately, because of its molecular structure, PPy is non-thermoplastic [27], mechanically rigid, brittle [36], and insoluble after synthesis [37]. PPy is now used in a wide range of applications, including microsurgical tools, biosensors, drug delivery, tissue engineering, neural probes, nerve-guidance channels, and blood conduits [36,37,38,39,40,41].

### 3.4. Polyaniline

Based on its oxidation state, polyaniline (PANI) refers to variable states of oxidation: fully oxidized state (pernigraniline base), half-oxidized state (emeraldine base), and fully reduced state (leucoemeraldine base). Among these, emaraldine base provides the most durable and conductive states [36,42]. PANI offers various benefits, including simplicity of fabrication, cheap cost, strong stability, and the ability to be electrically changed amongst conducting and resistive states [43,44,45,46,47]. Regrettably, its usage in microbial issues is displaying some restrictions because to its low process ability, poor flexibility, and non-degradability, and it has been reported to produce persistent inflammation after implantation in the body [20,44,48]. Several studies have been performed on the PANI and its application in neuronal probes, biosensors, tissue engineering, neuronal probes, and neuronal probes [49,50].

### 3.5. Polyurethane

Polyurethane (PU) is a polymer containing from sequence of urethane groups and has a vast of characteristics and chemical structures. Because of the variable mechanical and thermal properties and excellent biocompatibility of this polymer, PU biomaterials for prosthesis, wound dressings, artificial organs, vascular stents, and tissue engineering was rapidly developed in the last few decades. Numerous researchers all over the world are looking on expanding the applications of PU or to optimize its properties to meet the needs of particular application [51]. Biomaterials based on PU are non-immunogenic, resistant to most of chemicals in physiological fluid environments, nontoxic, and sometimes even biocidal [52,53]. PU are so adaptable, the hydrophobicity, hydrophilicity, biocompatibility, biodegradability, and capabilities to recombine with proteins, drugs, or biologically active substances that they possess can all be modified to meet the requirements of a broad range of applications through the use of a number of functional teams [54,55].

## 4. Polymer Nanocomposites

The successful fabrication of polymer nanocomposites is necessary for achieving high-performance, multifunctional next-generation materials. Duo to the distinguished characters of polymer matrix like flexibility, transparency, and light weight, the combination of them into nanofillers may promote the physico-chemical and mechanical characteristics of the nanocomposites [56,57,58,59,60,61,62,63,64,65]. A huge effort has been put to boost the physical performance of nanocomposites such as thermal stability and conductivity, electrical conductivity, isolation performance, etc. [66,67,68,69,70,71].

From the past decades, the improvement of structural and functional characteristics of the hybrid materials such as nanocomposites-based polymer-matrix have attracted the researcher’s attention. Because the combination between nanocomposites and polymers acting to promote the material characteristics better than polymers alone. The combination could be performed via organic and inorganic nanofillers with size down 100 nm and high surface area per volume [72]. Carbon nanotubes, silicates, metals, metal oxides, and ceramics are commonly used as nanofillers, which have different properties. In this regard, a fundamental understanding of nanostructures is essential to fabricate materials useful for the desired application. The functional and mechanical characteristics are the main changes of the new that produced from the combination between nanocomposites and polymer matrix, and the controlling of interaction and tuning other parameters, resulted on unique property combinations can be realized [73,74]. By taking advantage of the nanomaterials properties and selecting appropriate polymer matrices, a variety of nanocomposite materials have been prepared for different functional requirements [75]. Morphology, thermal and electrical properties was observed on composites such as poly (ethylene terephthalate) (PET) loaded with CNT, exfoliated graphite (EG) and hybrid materials as Mg(OH)_2_, where the addition of carbonaceous filler led to increase the crystallization and the charge transport of composites [76].

Polymer nanocomposites have incited a huge interest for different biomedical and biotechnological uses [77]. Research in this area is interdisciplinary, combining elements of materials science, nanotechnology, and biological science and leading to the design and development of more advanced materials [78,79]. These improvements are mainly seen in their mechanical properties. For example, polymers nanocomposites have been utilized to reproduce high-performance natural products such bone and silk [80]. Bioinspired materials are the materials that mimicking from the biological tissues. The mixing of soft polymer matrix with a hard nanostructure, resulting a bioinspired material. Novel fabricated composites are created by spreading hard immersion into a polymer matrix. Whilst, the main challenge that face the researchers when fabricate the bioinspired material is the equal dispersion of nanofillers into polymer matrix [81]. To overcome this challenge, different processing technologies have been employed [82]. Another key issue that must be solved is poor biocompatibility. For example, a Zn/Al layered double hydroxides (LDHs) was used in carbon nanotubes (80% of CNTs) and then added into a biodegradable highly amorphous vinyl alcohol polymer in order to improve the degree of dispersion of the filler into the polymer matrix [83].

In this review, we highlighted on the biomedical uses of polymer nanocomposites based on carbon nanotubes (CNTs), graphene and fullerene, highlighting their uses in tissue engineering and drug delivery, among other fields. Nowadays, the nanomaterials based on carbon are exponentially growth in the different industrial technology fields. A wide range of structure, shape, size, morphology, and dimension have been synthesized such as graphene, graphite, carbon nanotubes, activated carbons, etc. Graphite is the most common form of low-dimensional allotrope of carbon. Among this family of carbon nanomaterials, the sp2-carbon-based materials (carbon nanotubes, graphene, fullerenes, etc.) one of the best carbon nanomaterials that are using in the biomedical application and introduced in many medical operations like gene transfection, chemo-photothermal synergistic therapy, vivo real-time imaging, and drug delivery [84,85,86,87,88]. This may refer to their good characteristics such as high adsorption capability, photothermal conversion capacity, easy to manufacturing, and high compatibility in addition to their unique chemical, optical, and mechanical characteristics [89,90,91,92].

The improvement and the continuous enhancement of physico-chemical properties of the next generation of biomaterials also called CANOMATs specially carbon nanotube, have been took the interest of biomedical researchers because the good properties. These properties include hollow structures, good surface-area-to-volume ratios, high electrical conductance and thermal conductivity, mechanical stiffness, and the possibility to functionalize them in order to change the characteristics that are fundamental to them. The purpose of functionalization is to enhance both the solubility and biocompatibility of the substance in physiological settings. CANOMATs may be further conjugated with polymers, peptides, proteins, nucleic acids, and other kinds of biomolecules and medicinal agents in order to target certain varieties of cells, tissues, and organs [90].

## 5. Common Carbon Nano-Fillers

### 5.1. Graphene

In 2004 Geim and colleagues developed a graphene as a carbon material a two-dimensional (2D) framework. Since that time, graphene received a lot of awareness according to its important. Graphene contained from one atom broad planar sheets of sp2 carbon atoms in a honeycomb crystal lattice. This structure, along with the existence of free p-orbital and interaction sites for surface reactions, an aromatic form, and strong in-plane carbon-carbon bonding, creates a singular material with distinct mechanical, physicochemical, thermal, electrical, optical, and biological characteristics [93,94]. Numerous aspects of this material, such as mechanical stiffness, strength, elasticity, and electrical and thermal conductivity, are superior [95]. Graphene is the strongest and most flexible substance ever discovered, additionally, it is totally impenetrable. It also has extraordinarily high fundamental mobility and the highest thermal conductivity yet recorded. Because graphene has a countable number of characters, it might be desirable for use in biological applications. It is a great choice for drug administration due to its huge surface area, high chemical clarity, and simplicity of functionalization, and its distinctive mechanical properties alter its suitability for tissue engineering performance. Functionalized graphene may also be used in quick tests for a variety of biological compounds, including glucose, cholesterol, hemoglobin, and DNA. The extra lipophilicity of graphene may help it penetrate membrane barriers, which is another difficulty in medication administration. Nowadays, graphene nanoparticles have been introduced in various biomedical applications such as biosensing, gene delivery, cancer therapy, and tissue engineering [84].

### 5.2. Carbon Nanotubes (CNTs)

CNTs are rounded graphene sheet cylinders that are seamless and have superior mechanical, chemical, and physical capabilities [96,97]. CNTs (SWNTs or MWNTs) have numerous uses, including composite materials [98], nanoelectronics [99,100], field-effect emitters [101], and hydrogen storage [102]. Aiming to better understand the potential biological applications of carbon nanotubes, research was conducted over the past several years. This research was inspired by carbon nanotubes’ appealing and distinctive physical properties [103,104,105,106]. Furthermore, the suitable chemical or physical treatments of carbon materials may lead to different surface morphologies, which, in turn, may improve their dispersion and adhesion in the polymeric matrix [107].

Due to their quick electron transfer kinetics, extreme light weight, chemical inertness, high tensile strength, various antimicrobial properties, ability to function as protein transporters, and reactive functional groups, CNTs are more biocompatible than other carbon materials. Additionally, CNTs are semi-metallic and metallic conductive, making them more elegant for use in environmental monitoring, food treatment, and clinical diagnostics. Additionally, CNTs are utilized to cure cancer and have a significant impact on the sensors processing tools as a crucial component of the variable pathogenic bacteria detection. Many CNTs even have antibacterial properties [108,109].

### 5.3. Fullerenes

In biomedical applications like as medication delivery, active oxygen moieties quenching, targeted imaging, and tissue engineering, a variety of unique functionalized fullerenes and nanocarbons showed promise [108,109,110,111,112,113,114,115,116,117]. Due to unique physicochemical properties of fullerenes, which makes them suitable for photodynamic treatment and combating multidrug-resistant bacteria [118,119]. According to reports, fullerenes have the ability to localize within mitochondria and other cell compartments where free radicals are formed, giving them biological antioxidant capability [120]. Radical species can easily attack fullerenes because of their abundance of conjugated double bonds and lowest unoccupied molecular orbitals (LUMOs) that can accommodate an electron. Fullerenes can interact with a desired number of superoxide molecules that cannot be consumed in the process. Which considered a good scavenger for radicals. Due to biological properties like their distinctive genetic design and antioxidant action, fullerenes and their derivatives may be antiviral, making them intriguing for treating illnesses like the human immunodeficiency virus (HIV) [121].

## 6. Synthetic Polymers Based on Carbon Materials

Whereas many research findings in tissue engineering have associated with different nanocomposite scaffolds, the incorporation of bioactive frameworks and variable drugs has been shown to effectively recreate tissue. An electrospinning method was used to create nanocomposite PCL-modified scaffolds based on graphene oxide nanosheets and osteogenic drugs such as simvastatin and dexamethasone for enhancing the osteogenic differentiation of MSCs [122]. The GO reinforcements, the cell viability, drugs improved hydrophilicity, and osteogenic differentiation, are mainly responsible according to the findings. Furthermore, the GO nanosheets and osteogenic drugs have a synergistic effect on the PCL polymeric matrix. These drug-eluting nanocomposite scaffolds could be used to engineer bone tissue.

**Figure 2:**
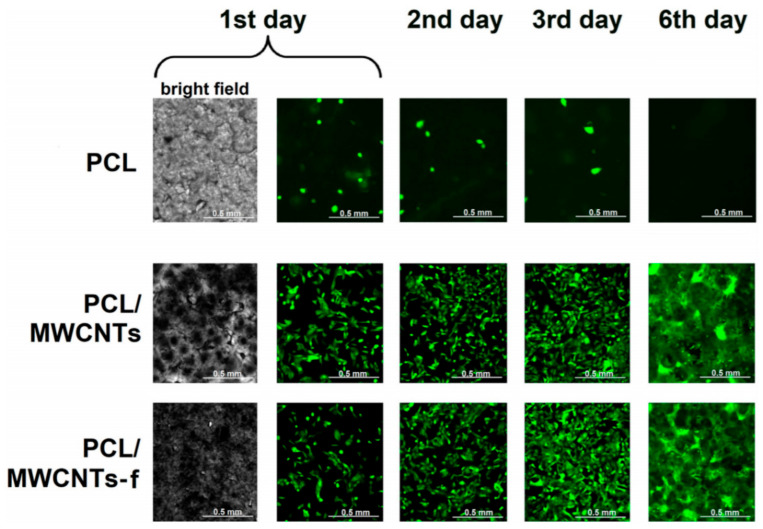
Human osteoblast-like fluorescence microphotographs U-2 OS-Green cells’ growth on tested nanomaterials [120].

Tissue engineering emerged years ago as a result of the discovery of new and advanced biomedical substitutes capable of replacing, maintaining, or even improving damaged tissue functions. CNTs are not toxic to human osteoblasts at low concentrations, according to research. More significant, Koodziej et al. prepared both of PCL/MWCNTs-f and PCL/MWCNTs by reinforced CNTs with polymer nanocomposites for biomedical application. They reported that the produced materials have better cell adhesion and allowing cells to grow on scaffolds [123]. To determine the nature of the cell–material interactions, they used 2D Raman correlation spectroscopy. In a cell proliferation assay, both substances were suitable to be biocompatible and osteo-inductive. Figure 2 displays fluorescence micrographs that showed the cell populations increase with time for both nanocomposite materials. As a result, the incorporation of CNTs is critical in improving the biocompatibility of these materials. In addition, the authors discovered that PCL/MWCNTs-f is more suitable for osteoblast adhesion and causes more progressing variations in the proteins of cultured cells than PCL/MWCNTs.

**Figure 3:**
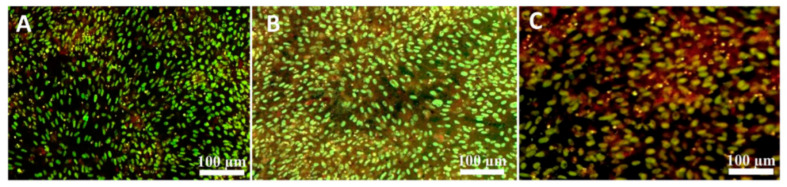
Saos-2 cell fluorescent images distribution on tissue culture plastic as a control (**A**); PCL-only scaffold (**B**); 1.0% weight MWCNT-PCL composite scaffold (**C**) [121].

Abdal-hay et al. [124] utilized air jet spinning (AJS) to create electrically conductive composite nanofibers of PCL and MWCNTs. AJS was used to overcome toxic effects that reduce the electrical conductivity of CNTs. Communications between PCL and MWCNTs improved both electrical conductivity and mechanical properties. Furthermore, in vitro studies revealed that these MWCNT-PCL nanocomposite fibers had good cell attachment and proliferation as shown in Figure 3.

**Figure 4:**
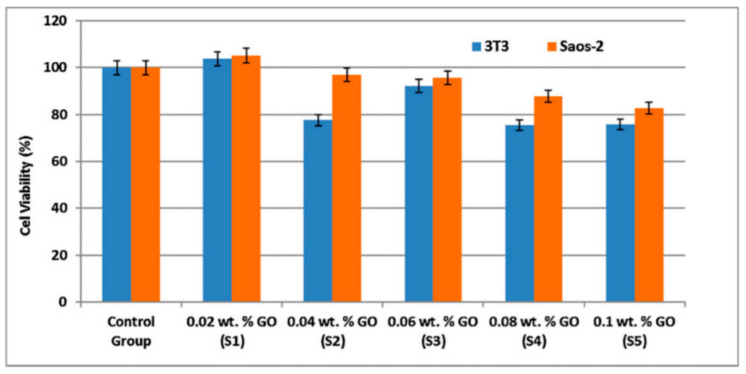
Schematic illustration for the MTT results of the living cells after 24 h [122].

Electrospinning was used to create PCL/GO/iron (II and III) oxide (Fe_3_O_4_) composite fibers that used as biocompatible scaffolds for biomedical uses [125]. This technique yields nanofiber with a high surface area, flexibility, and porosity. The above features contribute significantly to biocompatibility by determining cells with a familiar environment, resulting in improved and increased cell proliferation. The cell viability of the bioactive PCL/GO/Fe_3_O_4_ nanofibrous membranes decreased by up to 21.5 percent as the GO concentration increased as seen in Figure 4. The toxicity of graphene, on the other hand, is highly dependent on its oxygen contents and level of oxidation state, which are directly related to the synthesis techniques. Furthermore, it has been found that a low concentration of GO has no toxic effect on most cells. However, high levels of oxygen may cause cytotoxicity; thus, low concentrations of GO are appropriate for biomedical applications.

From another survey, 5 percent bioactive, PCL-coated glass scaffolds containing graphene nanopowder were intended and used in subchondral bone compartments using polymer foam study and dip-coating processes [126]. PCL scaffolds containing 10% by weight graphene were developed using a solvent casting and particulate leaching method and used to replace part of the osteochondral tissue in articular cartilage. All of the scaffolds contained porous structures that were connected to one another and electrically conductive. The biological response was measured in three dimensions using monoculture and co-culture structures. Under monoculture conditions, osteoblastic and chondrogenic cells had no toxic effect on the scaffolds, whereas co-culture medium resulted in higher levels of cell viability. The study demonstrates the scaffolds’ potential for osteochondral defect repair in bilayered osteochondral constructions. Different of GO/PCL nanocomposites have been synthesized as biocompatible materials, with different GO [126]. GO can be applied as a PCL modifier to enhance defects present in original PCL. Lomefloxacin (LMF) was utilized as a template drug, and the streaked release effects of GO/PCL and PLA-blended pills containing LM were investigated. The electrospinning procedure has been used to create biodegradable PCL nanofibers with varying concentrations of MWCNTs. The electrospun nanofibers were then efficiently ornamented using the shish-kebab shape using a crystallization technique (self-induced). CNT concentration influences fiber diameter and mechanical characters of electrospun nanofibers, as well as cell proliferation. Furthermore, the obtained shish-kebab decoration promotes the addition and proliferation of human osteogenic cells on the electrospun CNTs/PCL scaffolds, demonstrating their potential for bone tissue engineering [127].

Utilizing solvent casting and freezing methodologies, PCL, PEG, MWCNTs, and composite scaffolds based on 0.5% and 1% (*w*/*w*) MWCNTs plated by glue had been fabricated for cardiac tissue engineering [128]. The scaffolds’ variable characteristics including: mechanical, conductivity, degradation, contact angle, and sample cytotoxicity were all evaluated. The 1% (*w*/*w*) MWCNT scaffold with glue coating performed best overall, with good mechanical characters, good wettability, high electrical conductivity, adequate degradation, and an elegant respond to myoblasts. The mixing of MWCNTs to the PCL/PEG matrix resulted in higher in electric conductance. Graphene and MWCNT/PCL scaffolds were created using a casting and particulate leaching process for cartilaginous tissue engineering uses in the presence of suitable solvent [129]. The mixing of graphene or MWCNTs had no response on the porous network of the scaffolds, according to the results. Furthermore, both graphene and MWCNTs increased the tensile strength and electrical conductivity of the equipped PCL-based scaffolds at specified concentrations. The electrical conductivity of MWCNT/PCL composite was higher than that of G/PCL composites. As a result, the graphene and MWCNT/PCL-based scaffolds were thought to be promising materials for electrically stimulated cell growth. ACNT substratum was prepared using a plasma-enhanced chemical vapor deposition (PECVD) process further plated by PCL to improve the attachment of the cell and then recognize BMSCs into neurons [130].

The results showed that BMSCs on the PCL/CNTs substratum were successfully converted into neurons with the help of neuronal inducing factors. An explanation for the increase in cell adhesion as the PCL/CNT facilitates the adsorption of the protein. It was determined that the PCL/CNTs nanocomposite can be used as a substrate for engineering nerve tissues. Tohidlou et al. [131] studied the effect of amine-modified SWNTs (aSWNTs) onto the mechanical and chemical properties, as well as the bioactivity, of PCL scaffolds for bone tissue engineering. The addition of SWNTs improved the tensile strength while increasing the rates of bioactivity and degradation, according to the findings. The SWNTs additionally enhanced the PCL solution electrical conductivity, likely to result in a proper uniform size distribution of the thinner fibers. PCL/aSWNT scaffolds elucidated outstanding biological as well as mechanical properties upon comparing to pristine PCL scaffolds. Such findings point to the potential uses of PCL/SWNT electrospun scaffolds in bone tissue engineering.

It has been reported that the using of nanofiber-based drug delivery systems may having some side effects, also used frequently for the concern treatment. The electrospinning of fluorouracil (5FU) can create poly(-caprolactone)/poly(N-vinyl-2 pyrrolidone) (PVP) core–shell nanofibers filled with MWCNTs (PCL/PVP/MWCNT) loaded with 5FU [132]. The inclusion of MWCNTs in the shell augmented its tensile properties. Additionally, the increasing amount of PVP in the nanofibers improved degradability. On a line of cervical cancer cells, the drug-loaded nanofibers’ carrier non-toxicity and efficacy were confirmed. This phenomenon has the prospect to be utilized as a post-surgical drug delivery device for cancer treatment. Electrospinning techniques were used to create nanocomposite PU/PCL scaffolds/GO for potential skin tissue engineering [133]. The scaffolds were found to be biocompatible with skin fibroblast cells and could help in the design of skin tissue. Additionally, adding GO to the PU/PCL nanocomposite can enhance the biocompatibility and wettability of the scaffold.

Rikhari et al. [134] created a Ti metal PPy/GO composite coating for orthopaedic implants. They looked at how changing the amount of graphene oxide in the PPy matrix affected the results. The PPy/GO-coated Ti had adequate hardness, adhesion strength, and corrosion resistance. In vitro cell culture observation displayed that the PPy/GO composite had a better morphology and a higher proliferation rate of MG-63 cells than the PPy coating alone. Confocal images from in vitro MG-63 cell culture results with MTT assay data for Ti, PPy coating, and PPy/GO hybrid coated composite with Ti. The findings suggest that GO can connect with neighboring tissues without causing toxicity and can improve cell attachment and proliferation. PANI is an intriguing polymer due to its manageable chemicophysical features and antimicrobial properties in solution. Using ultrasonication and a frequently used during situ polymerization expriment in aqueous acidic solution, a new class of hybrid nanocomposites consisting of PANI, GNs, and CNTs were created for water disinfection. According to column chromatography, these new materials can remove *S. aureus* and *E. coli* from infected water [135]. Furthermore, research has shown that composite materials might recycled multiple times while employing nearly the same level of bacterial adsorption. The effectiveness of GN and CNTs in eliminating bacteria is refered primarily to their hydrophobic interactivities with the bacterial cell membrane, which promote phospholipid removal and oxidative stress via the assembly of reactive oxygen near the adsorbed cells, resulting in cell death. Because of its exceptional antibacterial properties, PPy is an appealing conducting polymer. The bioeffect of PPy is most likely due to its positive charge, which allows it to adhere to negatively charged bacteria on its surface and kill them. Pyrrole, silver nitrate, and SWNT composites were created through aqueous oxidative polymerization of pyrrole with silver nitrate [135]. Acolumn chromatography filter method for bacterial removal revealed that the CNT60/PPy/AgNPs nanocomposite was effective against *E. coli*, achieving 100% removal.

Wilson et al. [136] improved NADH oxidation applying polytyramine (PT)/MWCNT-altered electrodes for ethanol biosensing. They discovered that PT electrodeposition on MWCNT increased NADH oxidation by lowering the overpotential oxidation compared to Ag/AgCl. Because the lower oxidation potential reduces the risk of fouling electrodes and oxidizing enzymes, this electrocatalysis with immobilized NADH-based electrodes is advantageous for biosensing systems. To create composite materials with bigger and more stable conductivity for biological applications, GO nanosheets were used as a PANI dopant [135]. When compared to the non-nanoparticular PANI scaffold, all assays showed increased engagement of satellite cells towards cardiac lineage. While single satellite cells automatically differentiated into cardiomyocytes, cardiac gene markers as well as protein expression were greater using hybrid scaffold materials than their pure counterparts.

Singh et al. [137] created an efficient Fe_3_O_4_/MWCNT-/PANI/Nafion/GC electrode as an enzymatic type electrode for detecting urea in milk specimens. Such observation resulted in a better method for electrochemical urea sensing. An innovative electrochemical immune-sensor placed on palladium nanoparticles (PdNPs), PANI, and a fullerene-C60-modified glass carbon electrode (PdNP@PANI-C60 nanocomposite film/GCE) was developed for the exposure of biological markers for prostate specific antigens (PSA) [138]. The recommended immune-sensor demonstrated superior reply to PSA in serum and plasma samples, according to the results of the experiments. A simple investigation for the fabricated modified working electrode as a urea biosensor has been displayed in.

**Figure 5:**
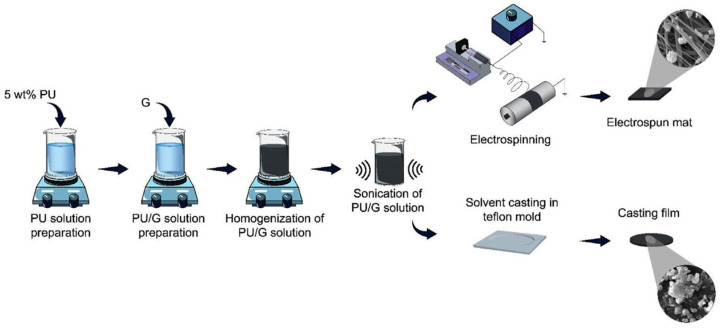
The fabrication techniques to create the PU/multilayer graphene flakes [136].

Tissue regeneration still enables the construction of outstanding intelligent biomaterials. Multiple studies on PU/G composites for biomedical engineering have recently been published. Bahrami et al. [139] used two separate fabrication techniques to create the PU/multilayer graphene flakes as membranes as shown in Figure 5: electrospinning and solvent casting. The results confirmed that electrospinning achieved a better distribution of graphene into the PU matrix than solvent casting. Cellular studies revealed that the PU/G composites increased cell adhesion and proliferation while remaining non-toxic. The existence of graphene on the surface of PU composites mended cell behavior. More particularly, graphene reduces fiber diameter, resulting in a bigger surface area for gripping proteins and more binding sites for cell membrane receptors. Graphene appeared to increase surface roughness, increasing the number of sites suitable for cell embedment. Sensors based on polymer-modified electrodes are used to quantify pharmaceuticals and related compounds. Because of its biocompatibility and good physical properties, polyurethane foam (PUF) is an important type of biopolymer.

Eshaghi et al. [140] recently developed a biosensor for the technique works best of the anticancer drugs capecitabine (CPT) and erlotinib hydrochloride (ETHC). The nanostructured sensor was built on the surface of PGE using MWCNTs and PU as a nanocomposite. The obtained date obviously demonstrated that the modified sensor is capable of detecting trace amounts of CPT and ETHC in real biological samples. Innovative biosensors three-dimensional scaffolds made of polyurethane foam and graphene oxide nanosheets have been developed as prospective three-dimensional scaffolds for the rehabilitation of skeletal tissue [141]. The impacts of GO on myogenic stimulation on skeletal myoblasts were also evaluated. Specifically, in 3D GO-PU foams, skeletal myoblasts’ cellular behaviour was evaluated using immunofluorescence analysis. The findings indicate that spontaneous myogenic differentiation by GO in the absence of myogenic factors was significantly encouraged, and the 3D GO-PU foams would display a proper 3D cell-growth microhabitat. Additionally, the 3D GO-PU foams’ myogenic stimulating effects improved spontaneous myogenic distinctiveness. This study revealed, 3D GO-PU foams can be employed to stimulate myogenesis and as biomimetic 3D scaffolds for the regeneration of lean tissue. Co-electrospinning DegraPolVR (DP), a polyester urethane, and various concentrations of GO solutions with polyethylene oxide resulted in 3D porous electrospun scaffolds (PEO) [142]. Electrospinning improved the elasticity of porous and fibroid scaffolds. The obtained date of such in vivo investigation disclosed scaffold decomposition, the absence of an exciting mechanism, and tissue cell invasion in the scaffold. Given these advantages, DP and DP/GO scaffolds are able materials for use in human and veterinary tissue engineering. Nanotechnology has favorable circumstances in the engineering of blood vessel tissue. The preparation of a multifunctional hybrid material in a scaffold form for vascular tissue engineering was investigated by combining the special mechanical, electrical, and biochemical characters of SWNTs with electrospun PU nanofibers [143]. Homogeneous dispersions and SWNT interactions with polyurethane chains refined the mechanical behavior of the composite, such as tensile strength and Young’s modulus, which may mimic the natural properties of the blood vessel. SWNT nanomaterials increased the melting temperature and replaced the melting character of electrospun nanofibrous scaffolds, according to the thermal properties. These findings imply that polyurethane nanofibers containing CNTs can mimic the biological performances of blood vessel extracellular matrix for vascular tissue engineering.

Altogether, the polymer nanocomposites are promising materials in different biomedical applications. Table 1 summarized the most recent carbon nanofillers together with possible polymeric matrices for significant number of biomedical applications as a versatile tool in this field.

## 7. Conclusions

A variety of hybrid nanocomposites for biomedical purposes have been created using variable carbon-based nanoparticles as nanofillers in polymeric materials. Several techniques have been used to reinforced the coupling between graphene, fullerene, and carbon nanotubes with polymer-based biomaterials such as dissolution, dispersion, and casting. In order to design the necessary variable types of hybrid composite materials, chemical modification techniques were used. Synthetic polymer matrices such as PANI, Ppy polymers, polycaprolactone, and polyurethanes have been used in the creation process. Fullerene, carbon nanotubes, and graphene have been commonly used as significant sources of carbon nanomaterials. Nanocarbons are improving significantly mechanical physical and chemical properties of polymers in the form of nanocomposites. Biologically relevant products were present in all of those bio-nanocomposites against the tested bacteria, fungus, and in vitro cell cultures.

## Figures and Tables

**Table 1:** An overview on the recent reported novel nanocomposites for a number of biomedical applications.

Composite Components	Applications	References
Functionalization of MWCNTs and polypyrrole loaded with the drug	Drug delivery system	[144]
Polycaprolactone/Graphene Oxide–Silver	Multi-biofunctional tissue scaffolds	[145]
Poly (vinyl alcohol)/chitosan/polyethylene glycol-assembled graphene oxide	Tissue engineering, wound dressing, and food-drug packaging industry.	[146]
Polycaprolactone/graphene oxide/strontium	Tissue engineering	[147]
Alginate/polycaprolactone/reduced graphene oxide	Skeletal muscle tissue engineering	[148]
Three-dimensional reduced graphene oxide/polyurethane scaffold	In vivo bone regeneration	[149]
Polyurethane/carbon nanotubes	Tissue engineering	[150]
Polyurethane/TiO_2_-MWCNT and Ag NPs	Tissue engineering	[151]

## Data Availability

All data generated or analyzed during this study are included in this review article.
